# Threat of Staphylococcus aureus Pneumonia in Severe COVID-19 Patients

**DOI:** 10.7759/cureus.22486

**Published:** 2022-02-22

**Authors:** Shuichi Tanaka, Koichiro Yamamoto, Hideharu Hagiya, Kou Hasegawa, Fumio Otsuka

**Affiliations:** 1 General Medicine, Okayama University Graduate School of Medicine, Dentistry and Pharmaceutical Sciences, Okayama, JPN

**Keywords:** ventilation, superinfection, pneumonia, staphylococcus aureus, coronavirus disease 2019

## Abstract

Coronavirus disease 2019 (COVID-19) has been spreading worldwide with unprecedented rapidity. *Staphylococcus aureus* is reported to frequently cause bacterial complications in patients with COVID-19. We herein present two additional cases of *S. aureus* pneumonia involving such patients. The first case was an obese 48-year-old man without any particular underlying diseases. The second case was another patient, a 72-year-old man, with hypertension, dyslipidemia, and steatohepatitis. Both patients developed methicillin-susceptible *S. aureus* pneumonia in the clinical course of COVID-19, to which antibiotic therapy with cefazolin was effectively administered. Through these cases, we emphasize that *S. aureus *secondary infections should be well cared with a high degree of caution in a case of critically ill COVID-19 patients.

## Introduction

Coronavirus disease 2019 (COVID-19), caused by severe acute respiratory syndrome coronavirus 2 (SARS-CoV-2), has been affecting the whole world.

Recent literature investigated the incidence of secondary bacterial infections in COVID-19 patients with mechanical ventilation due to acute respiratory distress syndrome [1]. Among 126 cases, 61% developed bacterial infections such as ventilator-associated pneumonia (VAP), blood stream infections, and urinary tract infections in its order, in which *Staphylococcus aureus* was the most common pathogen. Especially, methicillin-sensitive* S. aureus* (MSSA) accounted for 30% of the causative organisms of the respiratory infections. Another literature also suggested* S. aureus* to be the most common etiology of co-infection or superinfection secondary to COVID-19 [2]. We herein present two additional cases of MSSA pneumonia involving patients with severe COVID-19.

## Case presentation

**Figure 1:**
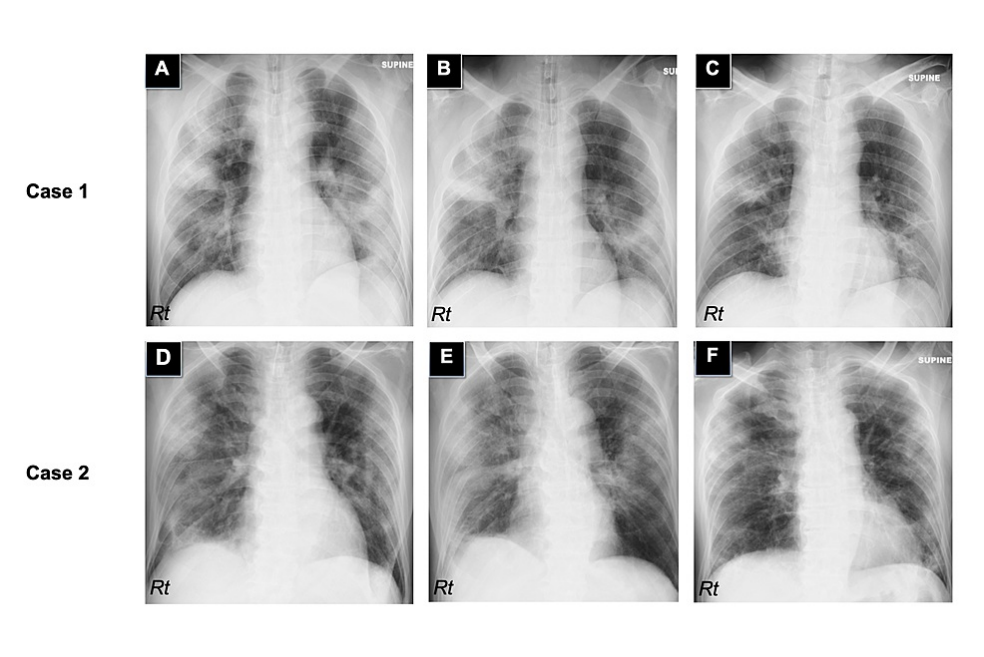
Imaging of methicillin-susceptible Staphylococcus aureus pneumonia in two COVID-19 patients. Chest X-rays of the first case on the fourth day of hospitalization (A), on the seventh day (B, before antibiotic therapy), and on the 16th day (C, after antibiotic therapy). Chest X-rays of the second case on the third day of hospitalization (D), on the sixth day (E, before antibiotic therapy), and on the 16th day (F, after antibiotic therapy). The infiltration shadows dramatically improved after antimicrobial treatment in both cases.

The first case was an obese 48-year-old man (body mass index, 30.6 kg/m^2^) without any other underlying diseases. The patient underwent the SARS-CoV-2 polymerase chain reaction (PCR) test and was diagnosed with COVID-19 in other institution before hospitalization in our hospital. On admission, his body temperature was 37.2°C and oxygen saturation level was 92% on 4 L/min oxygen supplementation. Laboratory tests revealed elevations of serum levels of C-reactive protein (CRP, 18.7 mg/dL), lactate dehydrogenase (608 U/L), and procalcitonin (0.44 ng/mL). Chest computed tomography showed bilateral multiple ground-glass opacities, which were compatible with COVID-19 pneumonia. The patient received 250 mg per day of methylprednisolone for three days in combination with 100 mg per day of remdesivir for five days. However, his respiratory condition deteriorated and the patient was intubated on the third day of hospitalization. Thereafter, his general status including the inflammatory markers and the findings on chest X-ray ameliorated (Figure 1A). On the seventh day of hospitalization, however, his sputum appeared purulent and the infiltrate shadows got worse (Figure 1B), accompanying elevated serum levels of CRP (16.3 mg/dL) and procalcitonin (0.21 ng/mL). Blood culture was negative. Sputum culture detected a large number of MSSA along with leukocyte migration, leading to the diagnosis of MSSA-induced pneumonia. We treated the patient with 6 g per day of cefazolin for 10 days. His respiratory condition subsequently ameliorated (Figure 1C), and the patient was discharged.

The second case was a 72-year-old man with hypertension, dyslipidemia, and steatohepatitis. The patient also had a confirmed positive result of SARS-CoV-2 PCR test in other hospital and was transferred to our hospital. Due to deteriorated respiratory condition (Figure 1D), the patient was intubated and administered with 125 mg per day of methylprednisolone for three days and a single dose of 8 mg per kg of tocilizumab. Subsequently, his respiratory status and serum inflammatory markers ameliorated. However, on the sixth day, the infiltrate shadows worsened (Figure 1E) and a purulent sputum emerged, which was positive for MSSA and active leukocytes. Blood culture resulted negative. We made a diagnosis of MSSA-induced pneumonia, to which antibiotic therapy with 6 g per day of cefazolin for 10 days was effectively administered. The patient recovered soon (Figure 1F), and was discharged. 

## Discussion

COVID-19 has been spreading worldwide with unprecedented rapidity. The disease potentially induces various complications, of which secondary bacterial infections should be regarded as the most harmful events [1]. Among various bacterial etiologies, recent literature indicated *S. aureus*-associated infections to be of great importance. For instance, an observational cohort study investigated the incidence of co-infections and superinfections in hospitalized patients with COVID-19 [2]. Overall, 74 (7.5%) of 989 patients had bacterial infection secondary to COVID-19, of which 44 cases (59.5%) were hospital-acquired superinfections complicating clinical course of patients with COVID-19. VAP accounted for 25% of the nosocomial infections (11/44 cases), in which *S. aureus* was reportedly the most common causative pathogen (36.4%). Another retrospective, single-facility study reported that 66 (5.52%) of 1,251 samples had bacterial infection secondary to COVID-19, of which* S. aureus* was reportedly the common causative pathogen (24.3%) [3]. Accordingly, bacterial complications, especially* S. aureus*-associated infections, do frequently occur in COVID-19 patients, although Gram-negative organisms, including *Pseudomonas aeruginosa* and *Acinetobacter baumanii*, are the representatives of VAP pathogens. Notably,* S. aureus* potentially causes various respiratory complications such as necrotizing pneumonia or pneumothorax [4], and, thus, clinicians should be aware of the clinical importance of prevention and management of such cases. 

When treating patients with *S. aureus* infections, an empirical coverage against anti-methicillin-resistant* S. aureus* (MRSA) is always an issue to be addressed. Clinicians should be aware of antibiogram, or MRSA isolation rate, at each medical situation, and determine the need of starting broad-spectrum antibiotics. In our hospital, the MRSA isolation rate for nosocomial cases changes approximately at 40-50% (data not shown). In the present cases, we carefully observed the patient’s clinical course without initiating anti-MRSA agents.

Antimicrobial stewardship should have a priority even for the patients with COVID-19. To differentiate deterioration of COVID-19 pneumonia and involvement of the secondary bacterial infections, procalcitonin would be of use [5]. Results of a retrospective, single-facility cohort study indicated that, based on a procalcitonin-guided algorithm, antimicrobial usages could be safely reduced in non-critically ill COVID-19 patients [6]. Emergence of antimicrobial resistance is another challenge that the global society is facing, which should be cared even amid the COVID-19 pandemic.

## Conclusions

We described two severe cases of *S. aureus* pneumonia involving ventilated patients suffering from COVID-19. *S. aureus* is usually not such a common pathogen in VAP. Compiling related literature with our cases, we would like to highlight that *S. aureus-*targeted treatment should be empirically initiated when COVID-19 patients manifest findings suggesting bacterial pneumonia.
